# Inequalities in Drug Access for Advanced Melanoma: The Prognostic Impact Resulting From the Approval Delay of the Combined Ipilimumab/Nivolumab Treatment in Portugal

**DOI:** 10.7759/cureus.78185

**Published:** 2025-01-29

**Authors:** Diana Baptista da Mata, Sara Coelho, Maria I Vilas Boas, Maria João Silva, Dânia Marques, Paula Ferreira

**Affiliations:** 1 Medical Oncology, Instituto Português de Oncologia do Porto, Porto, PRT

**Keywords:** advanced/metastatic melanoma, drug regulatory entities, immunotherapy, innovative therapies, targeted therapy

## Abstract

Introduction

A combination of ipilimumab/nivolumab has demonstrated a median overall survival (mOS) of 71.9 months in advanced melanoma, establishing it as the standard first-line (1L) therapy. However, the approval of this combination by the Portuguese Regulatory Authority occurred 76 months after its approval by the European Authority, leaving tyrosine kinase inhibitors as the only 1L option available for the BRAF-mutated melanoma population. Our study aims to evaluate real-world data from patients with advanced melanoma and assess the potential prognostic impact of the delayed availability of ipilimumab/nivolumab combination therapy on this population.

Methods

This was an observational, retrospective cohort study conducted at a Portuguese Comprehensive Cancer Center. The study included adult patients with melanoma who received innovative therapies in the 1L between May 2016 and December 2021 and who would meet the criteria for treatment with ipilimumab/nivolumab. The primary outcome measure was mOS; secondary outcome measures included median progression-free survival (mPFS), objective response rate (ORR), and safety data.

Results

Our study included 172 patients, of which 50% were male, and 32.6% (n = 56) had BRAF-mutated melanoma. In 1L setting, 70.9% received anti-programmed cell death protein 1 (anti-PD-1) monotherapy, while the rest were treated with targeted therapies. The median follow-up time was 57 months. Patients treated with anti-PD-1 had ORR of 36.0%, mPFS of seven months (95% CI 2.9-11.1), and mOS of 19 months (95% CI 7.5-30.4). Among patients treated with targeted therapies, the ORR was 56.0%, mPFS seven months (95% CI 5.1-8.9), and mOS 14 months (95% CI 5.9-22.1). In our population, 10% presented grade 3 or higher adverse events, with no drug-related deaths reported.

Conclusion

These findings underscore significant disparities in access to innovative therapies in Portugal, which may have adversely impacted patients' outcomes. The delay raises ethical concerns regarding equity in healthcare access and highlights the need for policy measures to expedite the approval and availability of life-extending treatments.

## Introduction

Malignant melanoma accounts for less than 10% of skin cancer cases [1]. Although only 4% are metastatic at diagnosis, its risk of recurrence at early stages and aggressive behavior make it the most frequent cause of skin cancer mortality [2,3]. The cutaneous form of melanoma is the most prevalent in the Western world [4], although it can also affect other organs such as the mucosa or choroid [5,6].

Melanoma is the 17th most common cancer worldwide, accounting for 302,570 cases in 2022, and the 22nd cause of cancer-related death [7]. The annual incidence in Portugal is 1.5% (with 1,215 cases diagnosed in 2022), placing melanoma as the 15th most frequently diagnosed cancer in Portugal and the 20th leading cause of cancer-related death [7].

The standard-of-care therapy of advanced melanoma has undergone significant changes in recent years, mainly due to the development of immunotherapy and molecular-targeted drugs [8]. These treatment advances have significantly altered the melanoma disease course, as shown by an increase in the overall survival (OS) and quality of life of these patients [9-11].

Targeted therapy acting against the v-raf murine sarcoma viral oncogene homolog B1/mitogen-activated extracellular signal-regulated kinase (BRAF-MEK) pathway was developed. As melanoma is frequently associated with oncogenic aberrations with BRAF mutations, drug development in the field of targeted therapies has arisen, first with the use of BRAF inhibitor (BRAFi) monotherapy (vemurafenib) in BRAF-mutated melanoma [6,12,13]. Nonetheless, multiple trials have demonstrated that the combination of a BRAFi and a MEK inhibitor (MEKi) had better results in this setting of patients when compared to vemurafenib in monotherapy. In this context, three combinations were approved in the palliative setting: vemurafenib with cobimetinib, dabrafenib with trametinib, and encorafenib with binimetinib [10,14-17].

In parallel with the development of targeted therapy, advancements in the understanding of tumor immunology and immune evasion have driven the development of numerous drugs [18,19], with interleukin-2 (IL-2) being the first immunotherapy treatment approved for advanced or metastatic melanoma [20]. Still, the high toxicity limited its use [20]. In 2011, ipilimumab, an anti-cytotoxic T-lymphocyte-associated protein 4 (anti-CTLA-4), was approved in this setting [4,14,18,19]. This drug has shown a beneficial impact on the median OS, which reached 10 months [21]. Anti-programmed cell death protein 1 (anti-PD-1) drugs were later approved, modulating the PD-1/PD-L1 complex and resulting in a greater anti-tumor response compared to ipilimumab monotherapy [9,11,12,18,19]. The first approved drug to act on this pathway was nivolumab [4]. This is a monoclonal anti-PD-1 antibody [4,14], which has shown survival rates of 62% at 12 months [4], with a positive impact in terms of progression-free survival (PFS) and OS in advanced melanoma [12,18,19,22]. Following the approval of nivolumab, pembrolizumab appeared as another anti-PD-1 monoclonal antibody [4,19,23], which also had a positive impact on the survival of patients with advanced or metastatic melanoma [11,12,22].

In 2015, the CheckMate 067 trial demonstrated improved outcomes in terms of OS (71.9 months) and PFS (11.5 months) for the combination of ipilimumab with nivolumab, becoming the standard of care in the first line (1L) setting for advanced or metastatic melanoma [9,12].

Drug approval timeline is presented in Appendix 1.

Although the evidence for the treatment options available for advanced melanoma is published globally at the same time, the approval times for the use of these drugs are highly dependent on the regulatory authorities. As such, the implementation of these drugs had different timings, depending on the date of approval of drug regulatory entities, such as the Food and Drug Administration (FDA) and the European Medicines Agency (EMA). Within the European Union, approvals are further dependent on national regulatory entities, leading to major disparities in access to these drugs. In Portugal, where approvals depend on the national authority Infarmed (the Portuguese acronym for the National Authority of Medicines and Health Products), these approvals may be delayed by years or, in some cases, may never occur, even if they have already been approved by the EMA. For instance, the approval of the use of immunotherapy combination treatment with nivolumab and ipilimumab by EMA happened eight months after the FDA’s approval [19]. However, Portugal had an additional 76-month approval delay, as the combination was only permitted by the Portuguese Regulatory Authority in September 2022 [24]. This resulted in a six-year delay in access to the most effective treatment available for the Portuguese population, compared with EMA’s approval.

Furthermore, in the BRAF-mutated melanoma population, anti-PD-1 monotherapy was also not approved, with BRAFi/MEKi being the only therapeutic option in the 1L setting [25]. Appendix 2 shows the differences between the European Society of Medical Oncology (ESMO) recommendations and the treatment availability in Portugal prior to the access to immunotherapy combination treatment.

In this study, we aimed to assess the impact of innovative therapies on advanced or metastatic melanoma, as well as the adverse effects on patients (either treated with 1L BRAFi/MEKi or immunotherapy monotherapy) who might have benefited from 1L treatment with the nivolumab/ipilimumab combination, if it had been available in Portugal since May 2016.

## Materials and methods

Patients

This study included adult patients with histologically confirmed melanoma of the skin, mucosal, or unknown primary site who started treatment with innovative therapies (immunotherapy or targeted therapy) in the 1L setting at a Portuguese Oncologic Comprehensive Center between May 2016 and December 2021. Follow-up was until July 2024.

Patients were excluded if they had an Eastern Cooperative Oncology Group (ECOG) 2 or higher, had a diagnosis of choroid melanoma, were included in clinical trials, had started systemic antineoplastic treatment in other institutions, or had received adjuvant treatment to melanoma. Patients with known brain metastasis at diagnosis were included if asymptomatic or previously submitted to brain surgery/radiotherapy with controlled brain metastasis and in need of a maximum daily dose of corticosteroid equivalent to oral prednisolone 10 mg.

Clinical, laboratory, and imaging data were collected through electronic patient records and reviewed by another investigator.

Study design

An observational retrospective cohort study of real-world data from a comprehensive cancer center. This study did not imply any changes in clinical practice and procedures currently occurring at IPO Porto. 

Endpoints

The primary endpoint was to evaluate the median OS of patients with advanced or metastatic melanoma and compare it to the available evidence of the randomized clinical trials that led to the approval of the innovative drugs, particularly the data of the CheckMate 067 trial. The secondary endpoints included assessing median PFS, ORR, and safety data. Exploratory analysis of prognostic factors impacting survival was also performed. 

Statistical analysis

Data were analyzed with descriptive and analytical statistics using the IBM SPSS Statistics for Windows (IBM Corp., Version 29, Armonk, NY).

Categorical variables were characterized through absolute and relative frequencies. Proportions were calculated for the number of patients from whom data were available. The numerical variables were characterized through measures of central tendency and dispersion such as mean, standard deviation, median, and interquartile range. Missing values were reported in the respective summary table. Only available data were summarized; no imputation methods were used to infer values from missing data, as the expected missing data were inferior to 5%.

ORR was defined as the rate of patients who had complete or partial response. PFS was defined as the time that occurred between the beginning of 1L treatment and the date of disease progression or death (whichever occurred first) or the end of follow-up. OS was defined as the time from the start of 1L treatment to death or the end of follow-up.

OS and PFS were calculated and presented graphically using Kaplan-Meier curves. The median OS and PFS were reported, as well as the respective 95% confidence interval (CI). The comparison of survival between groups of interest was carried out using the log-rank test. Exploratory analyses of potential prognostic factors influencing the OS were performed with univariate and multivariate Cox proportional hazard models. No correction for multiple hypothesis testing was established, as this analysis was exploratory and hypothesis-generating.

Ethics

This study was approved by the hospital administration and Ethics Committee (number CES/IPO: 03/023). The authors executed the necessary procedures to guarantee the anonymity and confidentiality of the obtained results.

## Results

Patients and treatment

**Figure 1:**
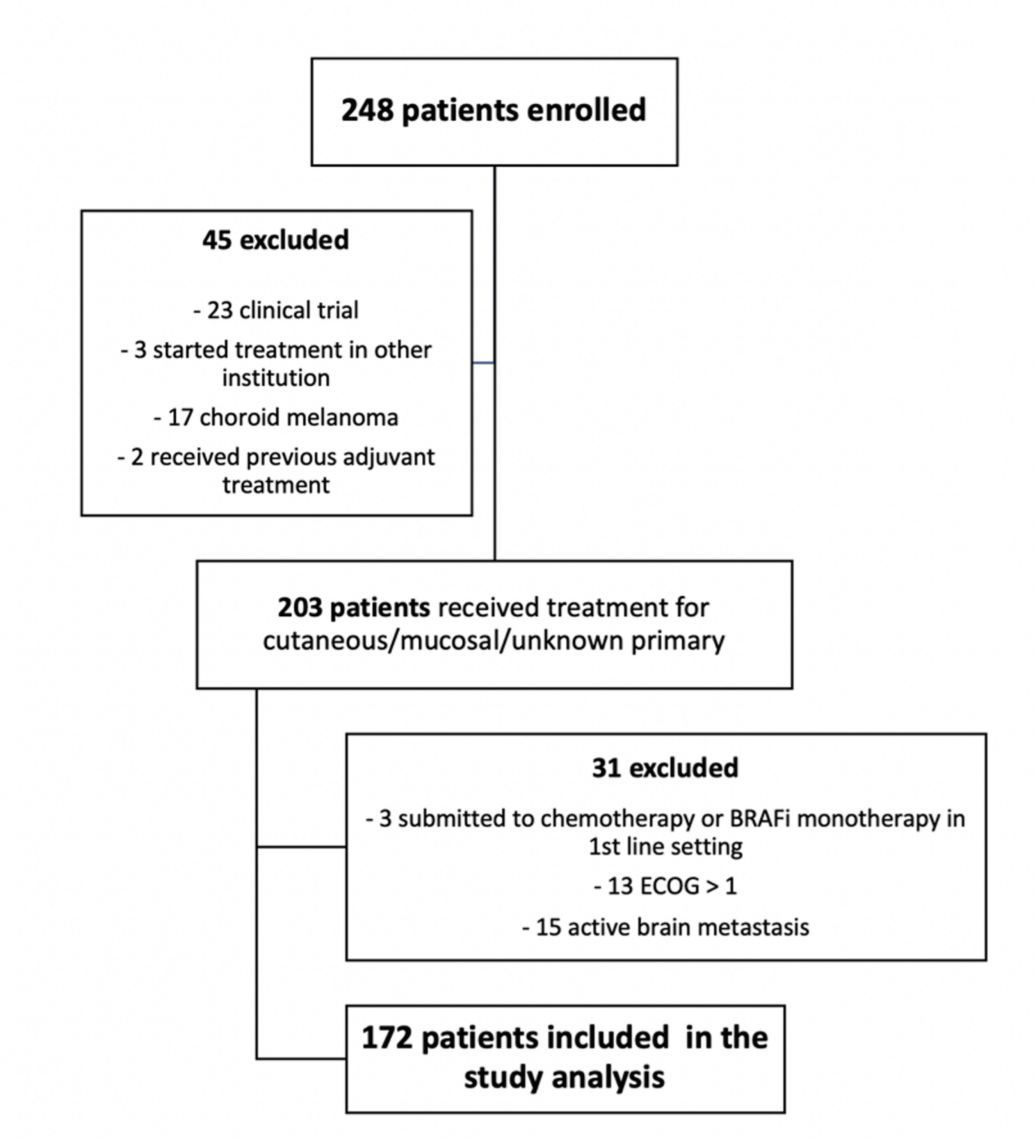
Flowchart with the patients included in this study.

Between May 2016 and September 2022, 248 patients with advanced melanoma were treated at our center with systemic antineoplastic therapy. After applying the exclusion criteria, 172 patients were included in our analysis (Figure 1).

The baseline characteristics of the population included in this study are detailed in Table 1. The median age was 69 (range 23 to 86) years, and half of the patients were male (n = 86, 50.0%). Most of the study population had a diagnosis of cutaneous melanoma (n = 149, 86.6%), followed by mucosal melanoma (n = 14, 8.1%) and unknown primary melanoma (n = 9, 5.2%). At the time of 1L treatment, 105 patients (61%) were classified as M1c, according to the American Joint Committee on Cancer (AJCC) seventh edition. Five patients (2.9%) were diagnosed with brain metastasis. Most patients had normal levels of lactate dehydrogenase (LDH), with approximately 35% (n = 61) showing elevated LDH levels. The BRAF status was evaluated in 170 patients (98.8%), with 32.2% having BRAF oncogenic mutations (n = 56; of those 89.3% BRAF V600E).

**Table 1:** Baseline characteristics of the study population AJCC - American Joint Committee on Cancer; ECOG - Eastern Cooperative Oncology Group

Characteristic	Value
Median age, year (range)	69 (23-86)
Sex, n (%)	
Male	86 (50.0%)
Female	86 (50.0%)
ECOG performance status, n (%)	
0	68 (39.5%)
1	104 (60.5%)
AJCC stage (seventh edition) at diagnosis, n (%)	
I	10 (5.8%)
II	33 (19.2%)
III	75 (43.6%)
IV	40 (23.3%)
Unknown	14 (8.1%)
Primary melanoma location at diagnosis, n (%)	
Cutaneous	149 (86.6%)
Trunk	12 (7.0%)
Dorsal	36 (20.9%)
Head and neck	16 (9.3%)
Upper limbs	11 (6.4%)
Lower limbs	34 (19.8%)
Abdomen	5 (2.9%)
Hands and feet	35 (20.3%)
Mucosal	14 (8.1%)
Unknown primary	9 (5.2%)
M stage at the time of first-line treatment (AJCC seventh edition), n (%)	
M0	3 (1.7%)
M1a	45 (26.2%)
M1b	24 (14.0%)
M1c	105 (61.0%)
Brain metastasis at the time of first-line treatment , n (%)	
Yes	5 (2.9%)
No	167 (97.1%)
Number of metastatic sites, n (%)	
<3	127 (73.8%)
≥3	42 (24.4%)
Type of metastasis involvement, n (%)	
Non-visceral	73 (42.4%)
Visceral	20 (11.6%)
Both visceral and non-visceral	76 (44.2%)
Lactate dehydrogenase at the time of metastatic disease, n (%)	
Normal	108 (62.8%)
Elevated	61 (35.5%)
Unknown	3 (1.7%)
BRAF status, n (%)	
Wild-type	114 (66.3%)
Mutation (V600E/V600K/V600G)	56 (32.6%)
Unknown	2 (1.2%)

Anti-PD-1 was the 1L treatment in 122 patients (70.9%) of the cases; of those, pembrolizumab was the treatment of choice in 69 patients (56.6%), and nivolumab in 53 patients (43.4%). Target therapy was used in 50 patients (29.1%) in 1L: the combination of dabrafenib with trametinib was the targeted therapy most frequently used (n = 39, 78.0%), followed by cobimetinib with vemurafenib (n = 7, 14.0%) and encorafenib with binimetinib (n = 4, 8.0%). 

1L treatment was suspended in 167 patients (97.1%), and of those, 120 patients (71.9%) were due to disease progression. In 16.3% (n = 28) of cases, treatment suspension resulted from a shared decision with the patient, and in 8.1% (n = 14), it was due to toxicity. In five cases, treatment suspension was due to death or worsening clinical status.

Of the patients who progressed, 10 (8.3%) had oligoprogression and were treated with surgery/radiotherapy for their progression. Second-line therapies were administered to 62 (37.1%) of the total patients, which corresponded to 51.7% of the patients who progressed, with ipilimumab being the most frequently used treatment (n = 26, 41.9%), followed by anti-PD-1 (n = 11, 17.7%) and anti-PD-1/anti-CTLA-4 combination treatment (n = 11, 17.7%). It is important to note that access to ipilimumab/nivolumab combination treatment in the second-line setting was available in a few selected cases, authorized through individual special permissions, prior to its approval by the Portuguese Regulatory Authority.

A subsequent third line was given to 5.2% of the total of patients (n = 9). Treatment lines are further detailed in Table 2.

**Table 2:** Treatment lines characteristics BRAF/MEK - v-raf murine sarcoma viral oncogene homolog B1/mitogen-activated extracellular signal-regulated kinase; BRAFi/MEKi - BRAF inhibitor/MEK inhibitor; ChT - chemotherapy; CTLA-4 - anti-cytotoxic T-lymphocyte associated protein 4; PD-1 - programmed cell death protein 1.

Treatment	Value
First-line treatment class (n, %)	-
Immunotherapy	122 (70.9%)
Anti-PD-1	122 (70.9%)
BRAFi/MEKi	50 (29.1%)
Second-line treatment class (n, %)	62 (37.1%)
BRAFi/MEKi	4 (6.5%)
Immunotherapy	48 (77.4%)
Anti-PD-1	11 (17.7%)
Anti-CTLA-4	26 (41.9%)
Anti-PD-1 and Anti-CTLA-4	11 (17.7%)
Chemotherapy	10 (16.1%)
Third-line treatment class (n, %)	9 (5.2%)
BRAFi/MEKi	7 (77.8%)
Immunotherapy	1 (11.1%)
Anti-CTLA-4	1 (11.1%)
Chemotherapy	1 (11.1%)

Efficacy of first-line therapy

The overall response rate to the 1L treatment was 41.9% (n = 72), irrespective of the type of treatment. The ORR was higher in the 1L setting for the patients treated with BRAFi/MEKi (n = 28, 56.0%) compared with anti-PD-1 (n = 44, 36.0%). There was also a lower proportion of progressive disease in BRAFi/MEKi treated patients (n = 9, 18.4%) compared to anti-PD-1 monotherapy (n = 46, 37.7%). Response rates according to 1L treatments are detailed in Table 3.

**Table 3:** Response to first-line treatment subgroup BRAF/MEK - v-raf murine sarcoma viral oncogene homolog B1/mitogen-activated extracellular signal-regulated kinase; BRAFi/MEKi - BRAF inhibitor/MEK inhibitor; PD-1 - programmed cell death protein 1.

Response rate, n (%)	First-line treatment	
	BRAFi/MEKi	Anti-PD-1
Complete response	11 (22.4%)	22 (18.0%)
Partial response	17 (34.7%)	22 (18.0%)
Stable disease	12 (24.5%)	32 (26.2%)
Progressive disease	9 (18.4%)	46 (37.7%)
Not evaluated	1 (0.6%)	0 (0.0%)

**Figure 2:**
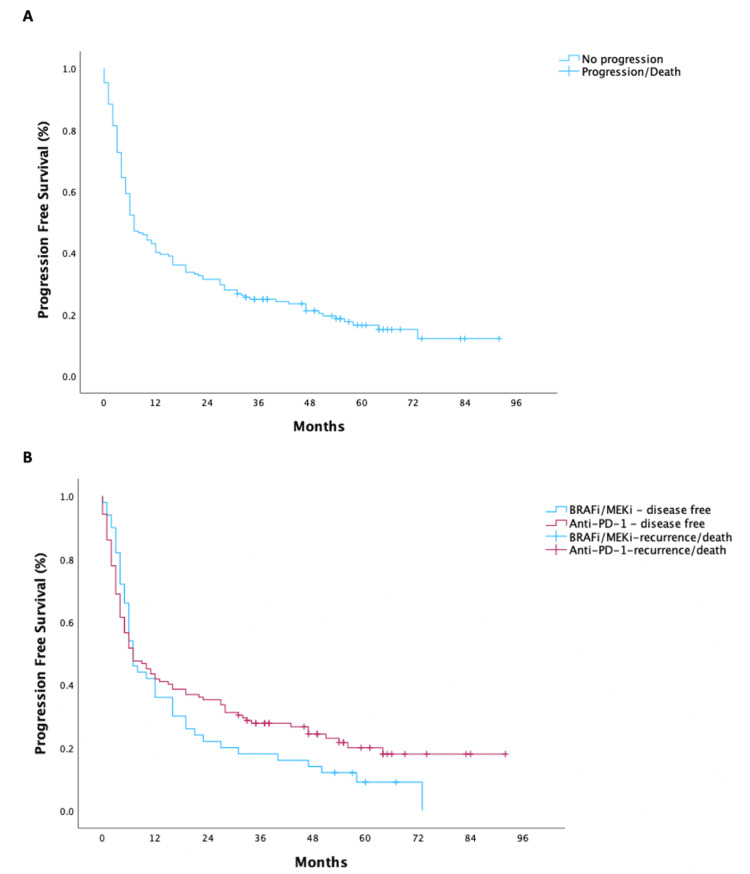
Progression-free survival curves: (A) progression-free survival for the population; (B) progression-free survival according to treatment type. BRAF/MEK - v-raf murine sarcoma viral oncogene homolog B1/mitogen-activated extracellular signal-regulated kinase; BRAFi/MEKi - BRAF inhibitor/MEK inhibitor; PD-1 - programmed cell death protein 1.

The median PFS in the global population of this study was 7.0 months (95% CI 4.5-9.5), as shown in Figure 2. 1L treatment with anti-PD-1 monotherapy mPFS was 7.0 months (95% CI 2.9-11.1), and with BRAFi/MEKi was 7.0 months (95% CI 5.1-8.9) (p = 0.313).

**Figure 3:**
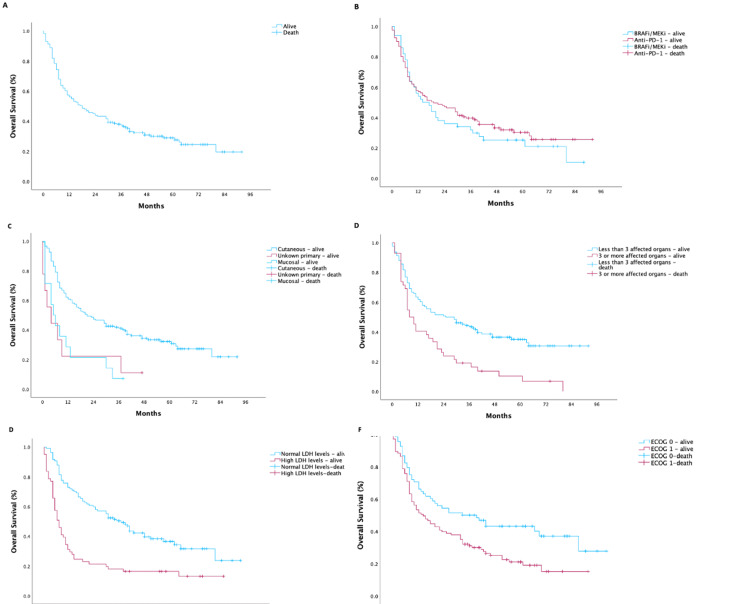
Overall survival curves: (A) overall survival for all the population; (B) overall survival according to treatment type; (C) overall survival according to melanoma type; (D) overall survival according to number of affected organs; (E) overall survival according to lactate dehydrogenase levels; (F) overall survival according to ECOG status. BRAF/MEK - v-raf murine sarcoma viral oncogene homolog B1/mitogen-activated extracellular signal-regulated kinase; BRAFi/MEKi - BRAF inhibitor/MEK inhibitor; ECOG - Eastern Cooperative Oncology Group; LDH - lactate dehydrogenase; PD-1 - programmed cell death protein 1.

The median OS in the global cohort was 18.0 months (95% CI 10.7-25.3), as illustrated in Figure 3. 1L treatment with anti-PD-1 monotherapy mOS was 19.0 months (95% CI 7.5-30.5) and with BRAFi/MEKi was 14 months (95% CI 5.9-22.1) (p = 0.447).

Exploratory analysis of prognostic factors impacting the OS was performed for ECOG, melanoma primary location, melanoma type, BRAF mutation status, M stage according to AJCC seventh edition, number of affected organs, LDH levels, and 1L treatment type. Of those, ECOG 0 (p = 0.017), cutaneous melanoma, p = 0.002), lower number of affected organs (p = 0.013), and normal LDH levels (p < 0.001) were identified as independent factors associated with better survival on multivariate Cox regression analysis. No statistically significant difference was observed based on BRAF mutation status (p = 0.846) or M stage according to AJCC seventh edition (p = 0.895). Figure 3 illustrates the OS curves according to the variables identified as associated with prognostic value.

Toxicities in first-line setting

Both immunotherapy monotherapy and BRAFi/MEKi were generally well tolerated, with less than 50% (n = 79) of any grade adverse events (AEs) reported. The most common toxicities associated with immunotherapy were cutaneous (n = 12, 7.0%) and endocrine (n = 21, 12.2%), while patients treated with BRAFi/MEKi experienced more frequently musculoskeletal (n = 9, 5.2%) and cutaneous (n = 8, 4.7%) AEs, in addition to pyrexia (n = 7, 4.1%), as described in Table 4. Grade 3 or higher AE occurred in 17 patients (9.9%), 6.6% (n = 8) with immunotherapy and 22.0% (n = 11) with BRAFi/MEKi. There were no treatment-related deaths. Only 14 patients (8.4%) discontinued treatment due to toxicity, nine (7.4%) with immunotherapy, and five (10.0%) with BRAFi/MEKi.

**Table 4:** Treatment-related adverse events BRAF/MEK - v-raf murine sarcoma viral oncogene homolog B1/mitogen-activated extracellular signal-regulated kinase; BRAFi/MEKi - BRAF inhibitor/MEK inhibitor; PD-1 - programmed cell death protein 1.

Event	Total		Immunotherapy		BRAFi/MEKi	
	Any grade, n (%)	Grade 3 or 4, n (%)	Any grade, n (%)	Grade 3 or 4, n (%)	Any grade, n (%)	Grade 3 or 4, n (%)
Gastrointestinal	16 (9.3%)	1 (0.6%)	8 (4.7%)	1 (0.6%)	8 (4.7%)	0 (0.0%)
Constitutional	8 (4.7%)	0 (0.0%)	1(0.6%)	0 (0.0%)	7 (4.1%)	0 (0.0%)
Cutaneous	20 (11.6%)	3 (1.7%)	12(7.0%)	0 (0.0%)	8 (4.7%)	3 (1.7%)
Pyrexia	7 (4.1%)	0 (0.0%)	0 (0.0%)	0 (0.0%)	7 (4.1%)	0 (0.0%)
Endocrine	21 (12.2%)	5 (2.9%)	21 (12.2%)	5 (2.9%)	0 (0.0%)	0 (0.0%)
Musculoskeletal	13 (7.6%)	0 (0.0%)	4 (2.3%)	0 (0.0%)	9(5.2%)	0 (0.0%)
Pulmonary	5 (2.9%)	4 (2.3%)	3 (1.7%)	2 (1.2%)	2 (1.2%)	2 (1.2%)
Renal	7 (4.1%)	1 (0.6%)	3 (1.7%)	0 (0.0%)	4 (2.3%)	1 (0.6%)
Liver	7 (4.1%)	3 (1.7%)	2 (1.2%)	0 (0.0%)	5 (2.9%)	3 (1.7%)
Ocular	2 (1.2%)	0 (0.0%)	0 (0.0%)	0 (0.0%)	2 (1.2%)	0 (0.0%)
Cardiac	2 (1.2%)	1 (0.6%)	0 (0.0%)	0 (0.0%)	2 (1.2%)	1 (0.6%)
Hematologic	2 (1.2%)	1 (0.6%)	0 (0.0%)	0 (0.0%)	2 (1.2%)	1 (0.6%)
Hemorrhagic	2 (1.2%)	0 (0.0%)	0 (0.0%)	0 (0.0%)	2 (1.2%)	0 (0.0%)
Neurological	0 (0.0%)	0 (0.0%)	0 (0.0%)	0 (0.0%)	0 (0.0%)	0 (0.0%)

## Discussion

This retrospective observational study highlighted the existence of disparities in access to oncological treatments across European countries, with a particular focus on melanoma patients being treated in Portugal. In the case of advanced melanoma, multiple barriers to accessing innovative therapies have been identified over recent years, emphasizing the persistent challenges faced by patients in benefiting from cutting-edge medical advancements.

Efficacy data in metastatic melanoma settings have shown significant differences between clinical trials and this real-world data study. There are a few reasons that can explain these different results, which can be attributed primarily to the inability to access the standard-of-care treatments in the first or subsequent lines.

In our population, we could only treat BRAF wild-type melanoma in the 1L setting with immunotherapy monotherapy (mainly anti-PD-1) and the BRAF-mutated melanoma with BRAFi/MEKi. Taking these limitations into consideration, our findings revealed that 70.9% of patients received anti-PD-1 therapy as 1L treatment, primarily with pembrolizumab and nivolumab. The overall response rate of the patients treated with anti-PD-1 in the 1L setting was 41.9%, with a rate of complete response of 18.0%, which aligns with the results of large anti-PD-1 monotherapy clinical trials demonstrating response rates of approximately 40% to 50% [9,11]. Also, 29.1% of patients were treated with BRAFi/MEKi in the 1L setting, and their overall response rate was 56.0%, with a rate of complete response of 22.4%, consistent with the results from clinical trials of these drugs [10,15-17]. These findings support the efficacy of these treatments regarding response rates in the real-world setting. 

However, regarding OS, our findings diverge from the clinical trial results. For the BRAF wild-type population, our data revealed a median PFS of 7.0 months and a median OS of 19.0 months. These results contrast with those from the clinical trial that led to the approval of immunotherapy monotherapy in this setting and are more visible when compared to CheckMate 067 [9,11]. When we examine the BRAF mutated population, we observe a median progression-free survival (mPFS) of 7.0 months and a median overall survival (mOS) of 14.0 months, respectively. However, clinical trials have shown even more divergent results, with mOS of 22 to 33 months for treatment with BRAFi/MEKi [15,17,23]. Again, the survival results of the CheckMate 067 study are even more discrepant [9]. It is also well established that treatment with anti-PD-1/anti-CTLA-4 combination in the 1L setting in the BRAF-mutated melanoma patients results in better outcomes when compared to the use of targeted therapy, as demonstrated in the SECOMBIT and DREAMseq trials [26,27].

The fact that response rates and PFS in our population are comparable to the clinical trials in the same setting and treatment context (although slightly inferior) and the OS is not may be attributed to further limitations to the subsequent line of systemic therapy in Portugal [15-17].

These limitations could explain the seemingly poorer survival outcomes compared with clinical trials when the first treatment was anti-PD1 or, more noticeably, when the first treatment was BRAFi/MEKi [15-17]. 

In fact, Portuguese patients with BRAF wild-type melanoma were unable to access the combination therapy (in the 1L or subsequently), limiting their treatment options to use anti-PD-1 and anti-CTLA-4 treatments separately. Furthermore, in the group of individuals with BRAF mutations, not only was there a limitation in receiving the ipilimumab/nivolumab combination, but prescribing anti-PD-1 medication alone was also not an option. This approach limited the treatment sequence administration to BRAFi/MEKi as the 1L treatment and anti-CTLA-4 as the second-line treatment.

Looking at the effectiveness data from CheckMate 067 (which demonstrated a mPFS of 11.5 months and a mOS of 71.9 months), it is evident that our results are unsatisfactory, indicating the possible negative impact on patients who have limited access to these treatments until 2022 [9]. 

The presence of adverse prognostic factors, such as elevated LDH levels and a higher number of affected organs, could have further exacerbated the challenges faced by patients who did not have access to combination therapies. Studies have shown that patients with high LDH levels and multiple metastases have poorer prognoses, which would likely have impacted the OS in our cohort [28].

Regarding safety, this study found that both treatments (immunotherapy alone or BRAFi/MEKi) were generally well tolerated. This information is widely known, as the frequency of grade 3 or higher AEs in clinical trials is typically below 10% for antiPD1 and below 60% for BRAFi/MEKi and manageable [11,15-17]. In our cohort, cutaneous and endocrine were the most common side effects linked to immunotherapy, while endocrine and pulmonary were deemed the most severe. Concerning the combination of BRAFi/MEKi, the main adverse effects that we found were issues related to the musculoskeletal system, skin, and gastrointestinal tract, with the most serious side effects affecting the liver, skin, and lungs. It is worth noting, however, that this is a retrospective study, with limitations inherent to data collection, and the rate of side effects may be underestimated, particularly if grade 1. The absence of treatment-related deaths and the low discontinuation rates due to toxicity (8.4%) reinforce the safety profiles of these therapies.

Nonetheless, when it comes to toxicities, particularly regarding anti-PD-1 monotherapy, our result is significantly lower compared to the reported toxicities. Furthermore, serious adverse advents (grade ≥ 3) exceeded 50% in the ipilimumab/nivolumab clinical trial [9], making the selection of the ideal patient profile for this combination a challenge. It could be interesting to establish an optimal patient profile with reduced toxicity risk and increased potential effectiveness from this immunotherapy combination.

While the data for all patients were carefully reviewed, resulting in a low percentage of missing information, this retrospective study has certain inherent limitations. One such limitation is the potential for patient selection bias, as the study population may include a higher number and greater severity of comorbidities compared to those in the clinical trials that led to the drugs' approval. To address this, we made an effort to include patients whose characteristics closely aligned with the inclusion and exclusion criteria of the CheckMate 067 trial. Additionally, due to the retrospective nature of the study, mild non-analytic AEs may have been underreported in the clinical records, highlighting a common challenge of retrospective analyses.

This study aimed to evaluate the impact that the delay in innovative therapies had on patients with advanced melanoma in a reference and high-volume Portuguese comprehensive cancer center. Above all, it underscores the need for equitable access to healthcare among European countries, particularly in the fight against cancer, in this case in the setting of advanced melanoma.

## Conclusions

Our study highlights the existence of several barriers to accessing innovative therapies for melanoma patients being treated in Portugal over the recent years.

As the treatment landscape continues to evolve, having ensured early access to ipilimumab and nivolumab combination for patients with advanced melanoma patients, could have substantially enhanced survival outcomes. Furthermore, developing predictive biomarkers to identify patients who would benefit most from combination therapy remains a crucial area of research.

## Figures and Tables

**Figure 4:**
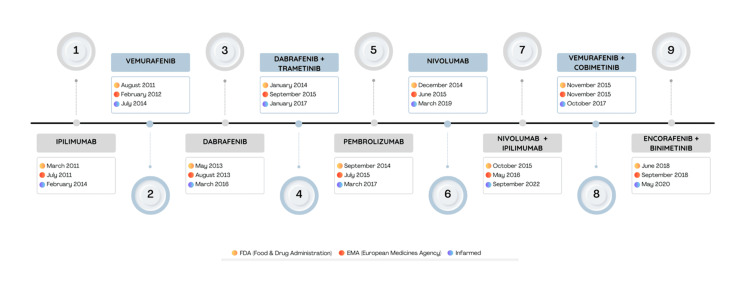
Drug approval timeline for FDA, EMA, and Infarmed (the Portuguese regulatory entity). EMA - European Medicines Agency

**Figure 5:**
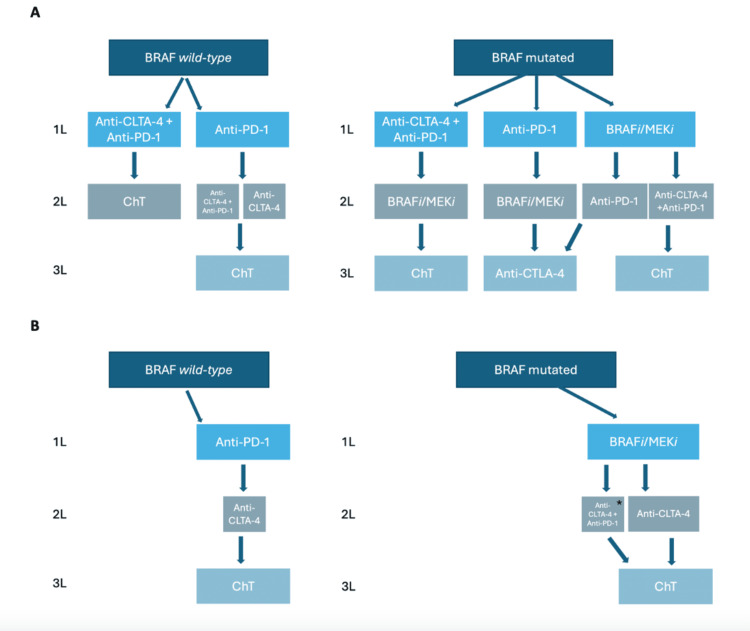
Differences between European Society of Medical Oncology (ESMO) recommendations (A) and the treatment availability in Portugal before the access to immunotherapy combination treatment (B). 1L - first line; 2L - second line; 3L - third line; BRAF/MEK - v-raf murine sarcoma viral oncogene homolog B1/mitogen-activated extracellular signal-regulated kinase; BRAFi/MEKi - BRAF inhibitor/MEK inhibitor; ChT - chemotherapy; CTLA-4 - anti-cytotoxic T-lymphocyte associated protein 4; PD-1 - programmed cell death protein 1 *Treatment combination access to ipilimumab/nivolumab in the second line was possible in a few selected cases before the approval of the Portuguese regulatory entity.

## Appendices

 Appendix 1

Appendix 2 

## References

[1] Michielin O, van Akkooi A, Lorigan P (2020). ESMO consensus conference recommendations on the management of locoregional melanoma: under the auspices of the ESMO Guidelines Committee. Ann Oncol.

[2] Tas F (2012). Metastatic behavior in melanoma: timing, pattern, survival, and influencing factors. J Oncol.

[3] World Health Organization (2024). Radiation: ultraviolet (UV) radiation and skin cancer. https://www.who.int/news-room/questions-and-answers/item/radiation-ultraviolet-(uv)-radiation-and-skin-cancer.

[4] Schadendorf D, Fisher DE, Garbe C (2015). Melanoma. Nat Rev Dis Primers.

[5] O'Sullivan DE, Boyne DJ, Gogna P, Brenner DR, Cheung WY (2023). Understanding real-world treatment patterns and clinical outcomes among metastatic melanoma patients in Alberta, Canada. Curr Oncol.

[6] Long GV, Swetter SM, Menzies AM, Gershenwald JE, Scolyer RA (2023). Cutaneous melanoma. Lancet.

[7] Ferlay J, Ervik M, Lam F (2024). Global Cancer Observatory: Cancer Today. https://gco.iarc.who.int/today.

[8] Ugurel S, Röhmel J, Ascierto PA (2017). Survival of patients with advanced metastatic melanoma: the impact of novel therapies-update 2017. Eur J Cancer.

[9] Wolchok JD, Chiarion-Sileni V, Rutkowski P (2025). Final, 10-year outcomes with nivolumab plus ipilimumab in advanced melanoma. N Engl J Med.

[10] Robert C, Karaszewska B, Schachter J (2015). Improved overall survival in melanoma with combined dabrafenib and trametinib. N Engl J Med.

[11] Robert C, Schachter J, Long GV (2015). Pembrolizumab versus ipilimumab in advanced melanoma. N Engl J Med.

[12] Michielin O, van Akkooi AC, Ascierto PA, Dummer R, Keilholz U (2019). Cutaneous melanoma: ESMO Clinical Practice Guidelines for diagnosis, treatment and follow-up†. Ann Oncol.

[13] Chapman PB, Hauschild A, Robert C (2011). Improved survival with vemurafenib in melanoma with BRAF V600E mutation. N Engl J Med.

[14] Skudalski L, Waldman R, Kerr PE, Grant-Kels JM (2022). Melanoma: an update on systemic therapies. J Am Acad Dermatol.

[15] Larkin J, Ascierto PA, Dréno B (2014). Combined vemurafenib and cobimetinib in BRAF-mutated melanoma. N Engl J Med.

[16] Robert C, Grob JJ, Stroyakovskiy D (2019). Five-year outcomes with dabrafenib plus trametinib in metastatic melanoma. N Engl J Med.

[17] Dummer R, Ascierto PA, Gogas HJ (2018). Encorafenib plus binimetinib versus vemurafenib or encorafenib in patients with BRAF-mutant melanoma (COLUMBUS): a multicentre, open-label, randomised phase 3 trial. The. Lancet Oncol.

[18] Finn L, Markovic SN, Joseph RW (2012). Therapy for metastatic melanoma: the past, present, and future. BMC Med.

[19] Chan PY, Corrie PG (2024). Curing stage IV melanoma: where have we been and where are we?. Am Soc Clin Oncol Educ Book.

[20] Atkins MB, Lotze MT, Dutcher JP (1999). High-dose recombinant interleukin 2 therapy for patients with metastatic melanoma: analysis of 270 patients treated between 1985 and 1993. J Clin Oncol.

[21] Hodi FS, O'Day SJ, McDermott DF (2010). Improved survival with ipilimumab in patients with metastatic melanoma. N Engl J Med.

[22] Moreira RS, Bicker J, Musicco F, Persichetti A, Pereira AM (2020). Anti-PD-1 immunotherapy in advanced metastatic melanoma: state of the art and future challenges. Life Sci.

[23] Maverakis E, Cornelius LA, Bowen GM (2015). Metastatic melanoma - a review of current and future treatment options. Acta Derm Venereol.

[24] Infarmed Infarmed (2024). Relatório público de avaliação - nivolumab em associação com ipilimumab para o tratamento do melanoma avançado (irressecável ou metastático) em adultos. https://www.infarmed.pt/documents/15786/3368817/Relatório+de+avaliação+de+financiamento+público+de+Opdivo+%28nivolumab%29/44842774-bcca-610b-3c81-5cf779efeef5.

[25] Infarmed Infarmed (2024). Relatório de avaliação prévia do medicamento para uso humano em meio hospitalar. https://www.infarmed.pt/documents/15786/2329793/Avaliação+prévia+à+aquisição+de+medicamento+para+uso+humano+em+meio+hospitalar+-++Keytruda+%28DCI+-+pembrolizumab%29/e2bbc398-a58a-4b63-89bf-bd3d56d2bb3f.

[26] Ascierto PA, Mandalà M, Ferrucci PF (2023). Sequencing of ipilimumab plus nivolumab and encorafenib plus binimetinib for untreated BRAF-mutated metastatic melanoma (SECOMBIT): a randomized, three-arm, open-label phase II trial. J Clin Oncol.

[27] Atkins MB, Lee SJ, Chmielowski B (2023). Combination dabrafenib and trametinib versus combination nivolumab and ipilimumab for patients with advanced BRAF-mutant melanoma: the DREAMseq trial-ECOG-ACRIN EA6134. J Clin Oncol.

[28] Palmer SR, Erickson LA, Ichetovkin I, Knauer DJ, Markovic SN (2011). Circulating serologic and molecular biomarkers in malignant melanoma. Mayo Clin Proc.

